# Analysis of shape and spatial interaction of synaptic vesicles using data from focused ion beam scanning electron microscopy (FIB-SEM)

**DOI:** 10.3389/fnana.2015.00116

**Published:** 2015-09-01

**Authors:** Mahdieh Khanmohammadi, Rasmus P. Waagepetersen, Jon Sporring

**Affiliations:** ^1^Computer Science Department, University of CopenhagenCopenhagen, Denmark; ^2^Department of Mathematical Sciences, Aalborg UniversityAalborg, Denmark

**Keywords:** synaptic vesicles, shape analysis, orientation analysis, three-dimensional point process, *K*-function, mark variogram

## Abstract

The spatial interactions of synaptic vesicles in synapses were assessed after a detailed characterization of size, shape, and orientation of the synaptic vesicles. We hypothesized that shape and orientation of the synaptic vesicles are influenced by their movement toward the active zone causing deviations from spherical shape and systematic trends in their orientation. We studied three-dimensional representations of synapses obtained by manual annotation of focused ion beam scanning electron microscopy (FIB-SEM) images of male mouse brain. The configurations of synaptic vesicles were regarded as marked point patterns, where the points are the centers of the vesicles, and the mark of a vesicle is given by its size, shape, and orientation characteristics. Statistics for marked point processes were employed to study spatial interactions between vesicles. We found that the synaptic vesicles in excitatory synapses appeared to be of oblate ellipsoid shape and in inhibitory synapses appeared to be of cigar ellipsoid shape, and followed a systematic pattern regarding their orientation toward the active zone. Moreover, there was strong evidence of spatial alignment in the orientations of pairs of synaptic vesicles, and of repulsion between them only in excitatory synapses, beyond that caused by their physical extent.

## 1. Introduction

There is extensive knowledge of how neurons communicate and how nerve signal transport from one neuron to another, (Li and Chin, 2003; Jahn, 2004; Jahn and Fasshauer, 2012). Upon arrival of an action potential at the nerve terminal, the pre-synaptic plasma membrane depolarizes and voltage-gated Ca^2+^ channels open; the initial rapid rise in intracellular Ca^2+^ triggers exocytosis of readily releasable synaptic vesicles at the active zone of the pre-synaptic membrane and release of their neurotransmitter into the synaptic cleft (Südhof, 1995; Rosenmund and Stevens, 1996). The reserve pool of synaptic vesicles becomes available for neurotransmitter release upon strong stimulation (Rizzoli and Betz, 2005); This pool may be mobilized to replenish the pool of readily releasable vesicles (Prekeris and Terrian, 1997; Cheung and Cousin, 2011). The synaptic vesicles movement have been observed in different synaptic preparations (Llinás et al., 1989; Koenig et al., 1993; Ryan and Smith, 1995; Henkel et al., 1996). Moreover, glutamate-containing vesicles have negative charges (Striegel et al., 2012), which could induce electrostatic repulsive interactions between them.

It is conceivable that the synaptic vesicle movement and their electrostatic repulsion could influence their shapes and their spatial distribution within the synapse. Using two-dimensional image data shape changes of the synaptic vesicles in epileptic rats were studied by Fischer and Langmeier (1980) and Hovorka et al. (1997). The dynamic of vesicle diffusion with the target membrane (vesicle-membrane interaction) was studied using total internal reflection-fluorescence correlation spectroscopy (Kyoung and Sheets, 2008).

Khanmohammadi et al. (2014) further used two-dimensional transmission electron microscopy (TEM) images to study spatial distributions of synaptic vesicle locations in samples from stressed and non-stressed rats. However, three-dimensional datasets would permit more precise analysis of physical and structural characteristics in biological samples. Three-dimensional spatial distribution of pyramidal neurons (Jafari-Mamaghani et al., 2010) and chemical synapses (Anton-Sanchez et al., 2014) have been studied using spatial point process techniques. Jafari-Mamaghani et al. (2010) applied Ripley's *K*-function to analyze interactions between pyramidal neurons. Anton-Sanchez et al. (2014) also used the *K*-function to explore the three-dimensional spatial distribution of chemical synapses in different layers of cerebral cortex.

Studying the structural characteristics and spatial interactions of synaptic vesicles in the central nervous system can lead to fundamental understandings of nerve communications. Shape and orientation of synaptic vesicles can impact the speed of their movement and their fusion with the active zone of the pre-synapse. Focused ion beam-scanning electron microscopy (FIB-SEM) (Stokes et al., 2006; Merchán-Pérez et al., 2009) is a technique from which shape and spatial interactions of vesicles may be studied in three dimensions. A salient feature of FIB-SEM imaging is isotropy, meaning that the data has the same resolution in all dimensions. By annotating each slice of the high-resolution FIB-SEM images, three-dimensional reconstructions of synapses and their vesicles can be obtained. The configurations of vesicles can be considered as a three-dimensional marked point pattern, with points and marks given by the vesicle centers and the associated size, shape, and orientation characteristics. This permits the use of statistical methods for marked point processes (Diggle, 2003; Illian et al., 2008; Baddeley, 2010; Ba et al., 2014) to study spatial interactions between vesicles.

The novel features of the current work are the following: we consider three-dimensional representations of synapses obtained from FIB-SEM images of adult mouse brains and propose a novel three-dimensional marked point process methods to study interactions between vesicles in terms of their locations, shape and orientation characteristics. A vast study of the shape and orientation characteristics of individual vesicles, and their interactions in terms of their locations, shape and orientation characteristics is carried out on the data at hand. Specifically we observe that (a) the synaptic vesicles near the active zone are ellipsoidal in shape (b) the orientations of the ellipsoidal synaptic vesicles depend on the direction to the active zone, and (c) the orientations of the vesicles are aligned.

## 2. Materials and methods

The data for our three-dimensional analyses were derived from publicly available FIB-SEM images of one healthy adult mouse brain using the technique explained by Knott et al. (2008). Herein, we used data from the CA1 hippocampus region of the model mouse brain, with a block size of approximately 5 × 10 × 7 μm corresponding to a 1065 × 2048 × 1536 pixel^3^ volume. The resolution of each voxel was approximately 5 × 5 × 5 nm. The data was provided as multipage TIF files, which were annotated in MATLAB (Mathworks, MA, USA).

We randomly selected five imperforated excitatory (asymmetric) synapses and one inhibitory (symmetric) synapse, which have perpendicular and slanted active zones with respect to the sectioning direction. This selection has been considered to avoid the artifacts produced by angle of tissue sectioning. The selected synapses have pre-synaptic neurons, which were extracted into enclosed blocks of size 425 × 535 × 350, 850 × 1000 × 305, 600 × 610 × 400, 600 × 680 × 340, 650 × 575 × 455, and 1750 × 1435 × 300 nm^3^, respectively.

**Figure 1:**
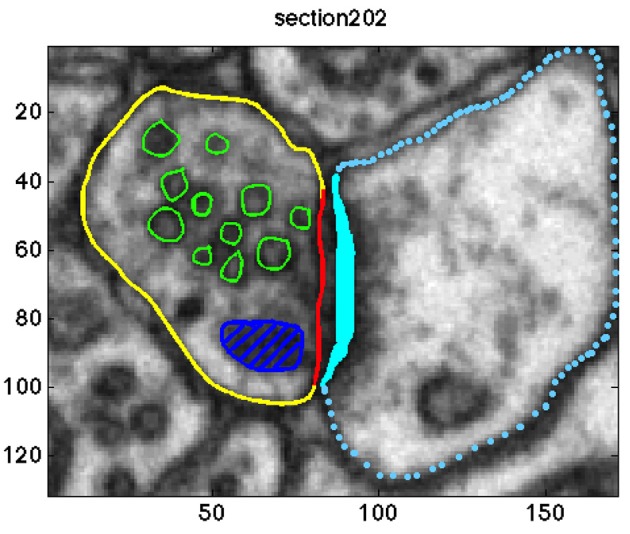
**Annotation of the objects of interests in one sample section of the data**. The synaptic vesicles, the mitochondria, the pre-synaptic membrane, the post-synaptic membrane, the active zone, and the post synaptic density (PSD) are shown in green, dark blue, yellow, blue, red, and cyan, respectively.

Overall, the pre-synaptic membranes of asymmetric synapses contained approximately 500 and the symmetric synapse included 131 synaptic vesicles. In a graphical user interface, an experimenter (MKh) annotated the objects of interest in each of the image sections. The following objects were segmented: the active zone, the boundary of each vesicle, pre-synaptic neuron, mitochondria, and lysosome. Since the synaptic vesicle diameter is approximately 40±5 nm (Qu et al., 2009), each synaptic vesicle appeared in 9–10 image sections. A sample of the annotations is shown in Figure 1.

### 2.1. Strategy for statistical analyses

We modeled synaptic vesicles as ellipsoids. For each vesicle the associated ellipsoid provides the best least squares fit of the boundary to the vesicle. Each ellipsoid is represented by its center and the vectors representing the axes of the ellipsoid (radii). For each ellipsoid the shortest straight path connecting the center and the active zone was found. We restricted our analysis to the point pattern of center coordinates and the sizes, shapes, and orientations of the ellipsoids.

We first conducted univariate analyses of the size, shape, and orientation characteristics of synaptic vesicles. We also consider the possible dependence of these characteristics and of the intensity of vesicle centers upon the distance to the active zone. Secondly, we used marked point process methods to study possible spatial interactions between neighboring vesicles regarding their positions and their marks defined as size, shape, and orientation characteristics. For this we employed a “hard core” null model, which permits vesicles to be located independently of each other except for the physical constraint that their associated ellipsoids are not allowed to overlap each other or intersect the membrane of the synapse, mitochondria, or lysosome inside the synapse.

## 3. Univariate analyses

In the univariate analyses we mainly focus on the shape of the vesicles and their orientation.

### 3.1. Size and shape

For an ellipsoid we let *a* > *b* > *c* denote the ellipsoid radii in decreasing order. The size of a vesicle is simply quantified by the approximate surface area $A\approx 4\pi {\left(\frac{{\left(ab\right)}^{1.6}+{\left(ac\right)}^{1.6}+{\left(bc\right)}^{1.6}}{3}\right)}^{\frac{1}{1.6}}$ of the associated ellipsoid (the approximation has a relative error smaller than 1.2%). Regarding shape we consider several size-independent shape characteristics:

1. *Elongation (aspect ratio)*: elongation $E=\frac{a}{c}$ is the ratio between the longest and the shortest radius. The elongation measures to which extent the vesicles are stretched. We also consider the ratios $\frac{a}{b}$ and $\frac{b}{c}$.
2. *Fractional anisotropy and mode of anisotropy*: the degree of anisotropy in the synaptic vesicles can be calculated by the fractional anisotropy (FA). It can be checked whether this fractional anisotropy is linear, orthotropic, or planar by measuring the mode of anisotropy (Westin et al., 1997; Ennis and Kindlmann, 2006). The fractional anisotropy is defined by $FA=\sqrt{3\mu_{2}/2(\mu_{1}{}^{2}+\mu_{2})}$, where μ_1_ and μ_2_ are the mean and variance of all radii of each ellipsoid. The mode of anisotropy is defined as $MO=\sqrt{2}\mu_{3}\mu_{2}{}^{-3/2}$, where μ_3_ is the third central moment of all radii of each ellipsoid. The mode of anisotropy can vary from −1 to 1 corresponding to a transition from planar anisotropy (*MO* = −1), to orthotropic (*MO* = 0), to linear anisotropy (*MO* = 1). The demonstration of the anisotropy space according to the fractional anisotropy and the mode of anisotropy is illustrated in Figure 2 in Ennis and Kindlmann (2006). Considering the fractional anisotropy and mode of anisotropy gives detailed insight regarding the shape of the synaptic vesicles.

### 3.2. Orientation

We assessed vesicle orientation relative to the active zone as a potential indicator of how they move toward it. For each vesicle we estimated the shortest straight path connecting the center of the vesicle to the active zone surface. Thereafter we calculate the smallest angles between the direction of this path and the three orientation vectors of the ellipsoid associated with the vesicle.

If the orientations of the vesicles are completely random, the distribution of the angles should be uniform on the interval from 0 to 90°. We conducted Kolmogorov–Smirnov tests to assess this hypothesis.

### 3.3. Dependence of vesicle density upon distance to the active zone

**Figure 2:**
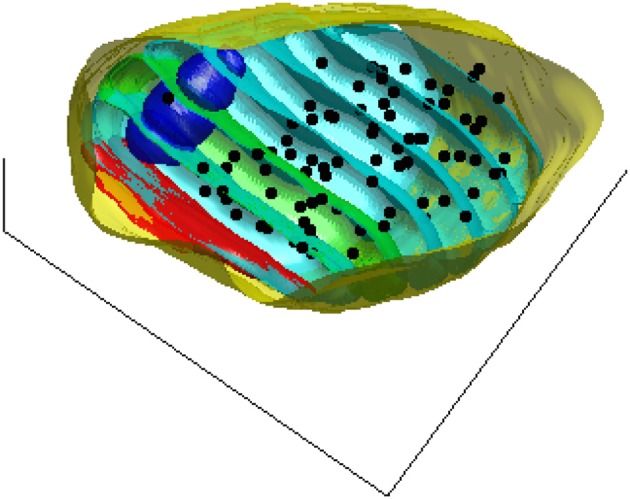
**A 3D view of the pre-synaptic compartments of one of the synapses including the active zone (red), the mitochondria (blue), the centers of the synaptic vesicles (black dots), and the slices (cyan) parallel to the active zone surface**. For better visualization one of the bands is shown in green.

To assess a possible dependence of vesicle density on distance to the active zone, we estimate the density of the synaptic vesicles in 50 nm thick contiguous slices parallel to the active zone. Figure 2 shows these slices for one of the synapses used in this study as well as the pre-synaptic compartment, the mitochondria, and the centers of the synaptic vesicles.

Slices were confined to the interior of the pre-synaptic membrane. The density for each slice was calculated as the number of the synaptic vesicles in each slice divided by the slice volume (excluding the volume occupied by any mitochondria and lysosome intersecting the slice).

We assess the dependency of the density on the distance from the active zone by fitting a generalized linear model to the counts of vesicles in each band. The expectation for the *i*th count was modeled as *E*[count_*i*_] = *V*_*i*_exp(α + β*d*_*i*_), where *V*_*i*_ is the volume of the *i*th band and *d*_*i*_ is the distance of the *i*th band to the active zone. To account for possible over- or under dispersion relative to the Poisson distribution, the default distribution for count data, we use the quasi-likelihood option available for inference in generalized linear models.

## 4. Interactions between vesicles

We investigated the electrostatic repulsion between the synaptic vesicles using techniques for spatial marked point processes, in particular variants of the *K*-function and the mark variogram (Møller and Waagepetersen, 2003; Diggle, 2003; Baddeley, 2010). Let *N* denote the number of vesicles, and let *N*_*i*_(*t*), *i* = 1, …, *N* denote the number of neighbor centers within distance *t* of the *i*th vesicle center. Let $\hat{\lambda }=N/V$ denote the observed intensity of vesicle centers, where *V* is the synapse volume. We then define an empirical *K*-function $\hat{K}(t)=\frac{1}{N\hat{\lambda }}\sum_{i}N_{i}\left(t\right)$ so that $\hat{\lambda }\hat{K}(t)$ is the empirical average of the *N*_*i*_(*t*). Thus, $\hat{K}(t)$ measures the tendency of points to form clusters or to repel each other. When *K*-functions are estimated from point pattern data, which is regarded as a partial observation of a larger point pattern, edge corrections are usually used (Møller and Waagepetersen, 2003). In our case, however, the centers are confined to the volume of the synapse and it does not make sense to assume that the pattern of vesicles extends outside the synapse. We therefore do not employ edge corrections. For better visualization we apply a simple one-to-one transformation of $\hat{K}$ into $\hat{L}$, which in the 3D case is given by $\hat{L}(t)={\left[\frac{3}{4\pi }\hat{K}(t)\right]}^{\frac{1}{3}}$.

To investigate the correlation between point process marks, like size or shape characteristics of neighboring vesicles, we consider, for a distance *t*, all the *N*_*t*_ pairs of centers with inter-center distance falling in an interval [*t* − Δ, *t* + Δ] around *t* for a Δ > 0. We compute the variogram *V*(*t*) of the marks associated with these centers:

$$V(t)=\frac{1}{N_{t}}\sum_{m,\;m^{\prime }}{\left(m-m^{\prime }\right)}^{2}$$

where the sum is over all pairs of marks associated with pairs of centers whose inter-center distance is between *t* − Δ and *t* + Δ. If there is no distance dependent interaction between marks, *V*(*t*) should be roughly constant. When looking at orientation interactions we replace (*m* − *m*′)^2^ with *v*(*m, m*′), where *m* and *m*′ in this case denote the corresponding main orientation vectors for a pair of vesicles, and *v*(*m, m*′) denotes the angle between these vectors.

It is evident that interaction between vesicles will be present due to the simple fact that vesicles can not overlap. To investigate whether there are interactions beyond those due to this, we compare the estimated point process characteristics for the observed data with estimates from simulations of the null model, which states that vesicles occur completely random except that their associated ellipsoids are not allowed to overlap with each other, the mitochondria, lysosomes, or the exterior of the synapse.

**Figure 3:**
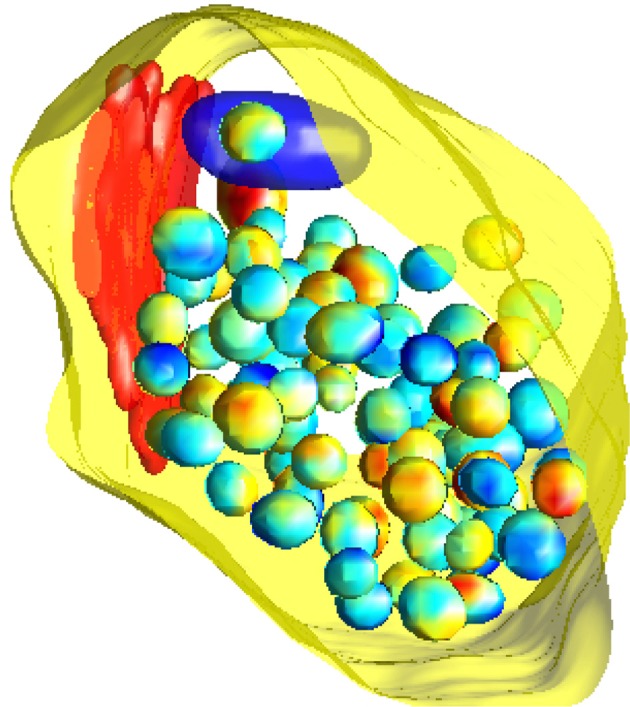
**A simulation of the null model**. The pre-synaptic membrane, active zone and mitochondria are shown in yellow, red, and dark blue, respectively. Vesicles are shown with interpolated colors, where the variation of color serves to better show the three-dimensional features.

To simulate from the null model, we use a birth-death Markov Chain Monte Carlo (MCMC) algorithm (Møller and Waagepetersen, 2003) initialized in the observed configuration of vesicles. Using the MCMC we generate a sample of 1000 random vesicle configurations, each of which can be considered as a simulation of the null model. An example configuration is shown in Figure 3. Note that our observation window for the MCMC approach is the highly complex shape of the state space for the vesicles given by the synapse excluding the mitochondria and the lysosome. Employing MCMC techniques for this irregular three-dimensional shape observation window is another novel feature of this study. We employ Ellipsoidal Toolbox (ET) for MATLAB to check whether the randomly generated ellipsoids overlap. This toolbox uses YALMIP and SeDuMi as an interface for the optimization tool (Löfberg, 2004; Henrion et al., 2012).

## 5. Results

**Figure 4:**
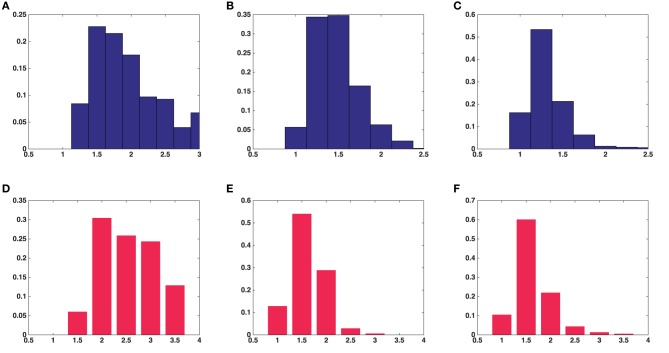
**Histograms of univariate shape characteristics of all the synaptic vesicles included in this study**. **(A)** Aspect ratio(*a*/*c*) in average over five asymmetric synapses. **(B)** Aspect ratio(*b*/*c*) in average over five asymmetric synapses. **(C)** Aspect ratio(*a*/*b*) in average over five asymmetric synapses. **(D)** Aspect ratio(*a*/*c*) in a symmetric synapse. **(E)** Aspect ratio(*b*/*c*) in a symmetric synapse. **(F)** Aspect ratio(*a*/*b*) in a symmetric synapse.

Results were obtained both for vesicles in each synapse separately and for all around 500 vesicles pooled together for asymmetric synapses. The second column in Table 1 shows that the mean surface areas of the synaptic vesicles are similar in all synapses. The histograms of shape characteristics in Figure 4 further show that the vesicles are far from spherical. Table 1 shows that the mean *a*/*b* ratio and mean *b*/*c* ratio appear to be considerably smaller than the mean *a*/*c* ratio for all asymmetric synapses. This also holds for the mean ratios for all vesicles considered jointly. These findings indicate that vesicles are of oblate ellipsoidal shape in the asymmetric synapses. On the other hand, in symmetric synapses the mean *a*/*b* ratio and mean *b*/*c* ratio appear to be very similar and considerably smaller than the mean *a*/*c* ratio. This indicates that the synaptic vesicles are of cigar ellipsoidal shape in the symmetric synapse.

**Table 1:** **Mean values of surface area and aspect ratios of synaptic vesicles for each asymmetric (AS1–AS5) and symmetric (SS1) synapses**.

**Synapse**	**Surface area (nm^2^)**	**a/c**	**b/c**	**a/b**
AS1	6592	1.5802	1.3559	1.1689
AS2	7831	1.7019	1.3243	1.2808
AS3	8785	2.2379	1.6272	1.3862
AS4	7637	2.2617	1.6180	1.3958
AS5	7931	2.0926	1.5358	1.3628
Average	7680	1.9590	1.4801	1.3176
SS1	8525	2.5958	1.6376	1.6007

*The average measurements for asymmetric synapses are also shown in the table*.

**Figure 5:**
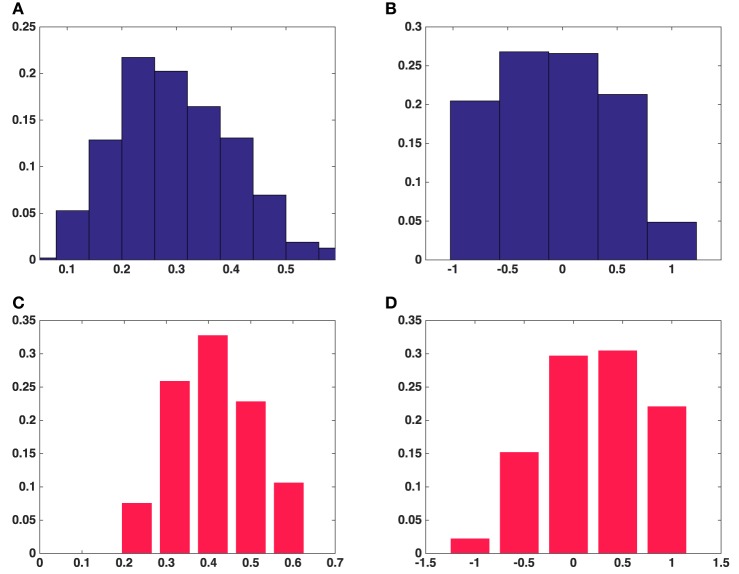
**Histograms of fractional anisotropy and mode of anisotropy of all the synaptic vesicles included in this study**. **(A)** Fractional anisotropy in five asymmetric synapses. **(B)** Mode of anisotropy in five asymmetric synapses. **(C)** Fractional anisotropy in a symmetric synapse. **(D)** Mode of anisotropy in a symmetric synapse. **(A,B)** Show the average fractional anisotropy and mode of anisotropy of synaptic vesicles in five asymmetric synapses, respectively. **(C,D)** Show the same measurements for synaptic vesicles in a symmetric synapse.

The histogram of the fractional anisotropy in Figures 5A,C illustrates that synaptic vesicles are Figure 5B indicates that this anisotropy varies across the range from planar to orthotropic and linear, but the tendency is toward the planar case for vesicles from the asymmetric synapses and the vesicles are of oblate shape. Figure 5D shows that the tendency of the anisotropy is toward linear case for vesicles from the symmetric synapse and the vesicles are of cigar shape.

**Figure 6:**
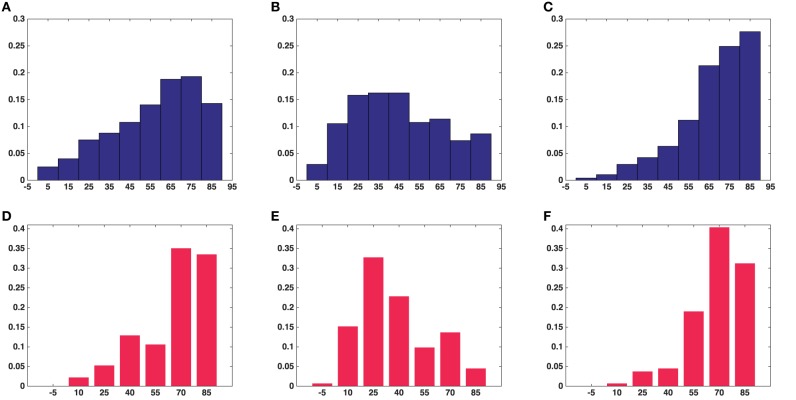
**Histograms of angles between the shortest straight path to the active zone and each of the orientation vectors of the ellipsoids in the all synapses**. **(A)** Angle with long axis (asymmetric synapses). **(B)** Angle with middle axis (asymmetric synapses). **(C)** Angle with short axis (asymmetric synapses). **(D)** Angle with long axis (symmetric synapse). **(E)** Angle with middle axis (symmetric synapse). **(F)** Angle with short axis (symmetric synapse). **(A–C)** Show the average measure for synaptic vesicles in five asymmetric synapses. **(D–F)** Show the measure for synaptic vesicles in a symmetric synapse.

Figure 6 shows histograms of the calculated smallest angles between the direction of shortest path to the active zone and the three orientation vectors of the ellipsoids associated with the vesicles. Kolmogorov–Smirnov tests provided *p*-values, represented in Table 2, for the angles between the shortest straight path to the active zone and, respectively the longest, middle, and the shortest orientation vector. This shows that the orientations are not random. In particular, the histogram for the shortest orientation vector shows that the angles between this vector and the direction to the active zone are in general close to 90° in asymmetric synapses. In the analyzed symmetric synapse, the histograms for the shortest and longest orientation vector show that the angles between these vectors and the direction to the active zone are in general close to 90°. The dependence of the density of synaptic vesicles on the distance to the active zone was not significant.

**Table 2:** ***P*-values of Kolmogorov–Smirnov tests for angle between the direction to the active zone and the long, middle, and short axes of the ellipsoids in five asymmetric synapses (AS1–AS5), in all of them pooled together, and in one symmetric synapse (SS1)**.

**Synapse**	***P*-value for angle with long axis**	***P*-value for angle with middle axis**	***P*-value for angle with short axis**
AS1	3.676 × 10^−2^	3.686 × 10^−3^	1.117 × 10^−20^
AS2	8.855 × 10^−5^	1.082 × 10^−1^	6.423 × 10^−26^
AS3	4.485 × 10^−7^	3.751 × 10^−2^	8.492 × 10^−8^
AS4	4.485 × 10^−7^	1.561 × 10^−5^	2.891 × 10^−21^
AS5	5.815 × 10^−12^	9.745 × 10^−3^	1.124 × 10^−7^
Average	3.897 × 10^−18^	9.017 × 10^−5^	4.924 × 10^−74^
SS1	3.781 × 10^−19^	2.763 × 10^−5^	7.094 × 10^−26^

**Figure 7:**
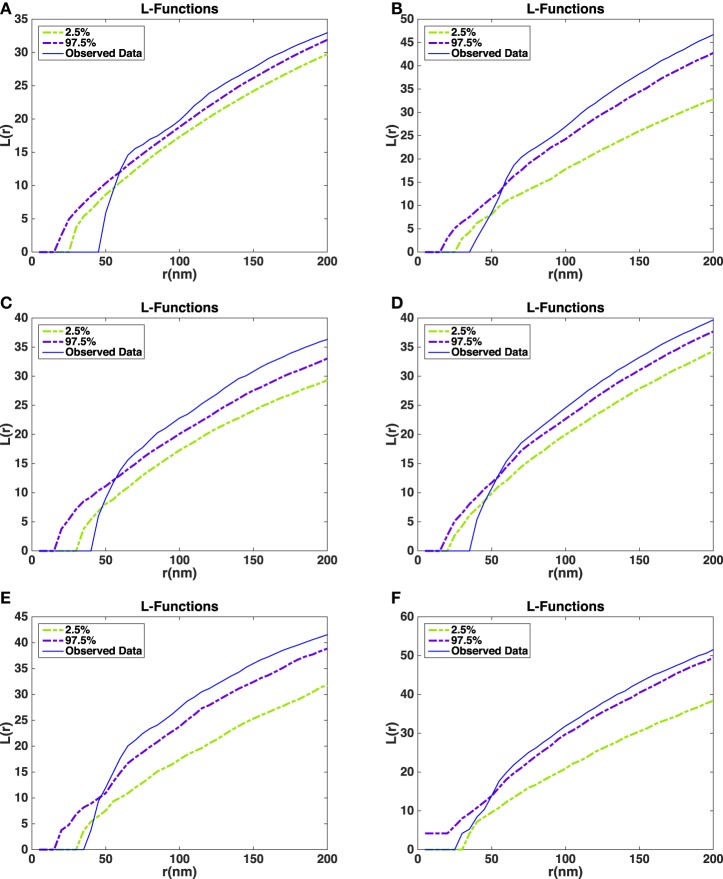
**Estimated *L*-functions for the five synapses**. **(A)** *L*-function asymmetric synapse 1. **(B)** *L*-function asymmetric synapse 2. **(C)** *L*-function asymmetric synapse 3. **(D)** *L*-function asymmetric synapse 4. **(E)** *L*-function asymmetric synapse 5. **(F)** *L*-function symmetric synapse 1. Dashed curves show point-wise 2.5 and 97.5% quantiles for the distribution of the *L*-function under the null model. Blue curves show the *L*-function of the observed data in the five asymmetric synapses **(A–E)** and a symmetric synapse **(F)**.

The *L*-functions for all of the synapses are shown in Figure 7. For each *t* the plot also shows point-wise 2.5 and 97.5% quantiles for the distribution of $\hat{L}(t)$ under the null model. The quantiles are obtained from the simulations of the null model.

The *L*-function for the asymmetric synapses falls below the 2.5% quantiles under the null model at distances up to 50 nm. This indicates that the repulsion between the vesicles is stronger than repulsion just caused by non-overlap. On the other hand, for large distances the estimated *L*-function is above the 97.5% quantile under the null model. This could be a sign of aggregation of vesicles at a larger scale possibly due to the reserve pool of vesicles (Rizzoli and Betz, 2005). In the symmetric synapse *L*-function for the data falls within 2.5 and 97.5% quantiles under the null model at distances up to 50 nm. This shows that there is no repulsion between the vesicles beyond the one caused by non-overlap. At larger distances the estimated *L*-function shows a similar behavior as in asymmetric synapses.

**Figure 8:**
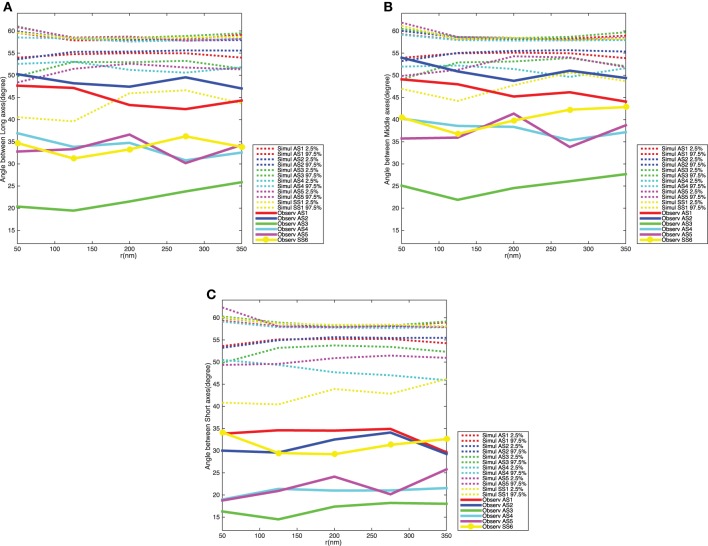
**Variogram of the synaptic vesicles orientation for each synapse**. **(A)** Angle between long axes. **(B)** Angle between middle axes. **(C)** Angle between short axes. The dotted curves show point-wise 2.5 and 97.5% quantiles for the distribution of *V*_*m*_(*t*) for the five synapses, while the red, blue, green, cyan, and magenta solid curves indicate the observed variograms for the five asymmetric synapses and yellow solid curve with dots shows the observed variograms for one symmetric synapse.

Figure 8 shows the variograms for the orientation of the synaptic vesicles together with 2.5 and 97.5% quantiles for the distribution of the variogram under the null model. The variograms fall well below the 2.5% quantiles, which shows that the average angles between orientation vectors for pairs of vesicles are much smaller than under the null model. The variograms are further approximately constant of values, this shows that the ensemble of synaptic vesicles is aligned with respect to the orientations of the vesicles. The difference between the distribution of the variogram under the null model and the variogram for the data is clearest for the short axes of the neighboring synaptic vesicles. This agrees well with the results of the analysis of vesicle orientations in relation to direction of the shortest straight path to the active zone.

**Figure 9:**
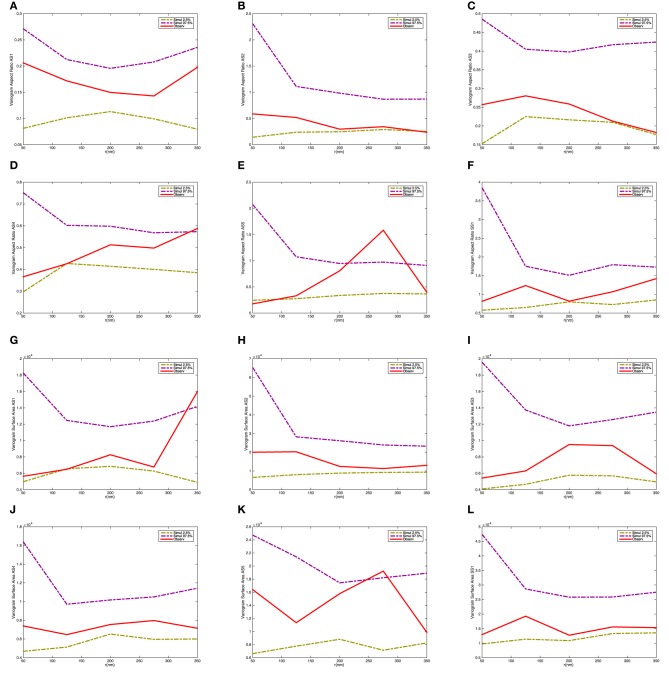
**Variogram of the synaptic vesicle size and aspect ratio for each synapse**. **(A)** Variogram of aspect ratio *a*/*c* for AS1. **(B)** Variogram of aspect ratio *a*/*c* for AS2. **(C)** Variogram of aspect ratio *a*/*c* for AS3. **(D)** Variogram of aspect ratio *a*/*c* for AS4. **(E)** Variogram of aspect ratio *a*/*c* for AS5. **(F)** Variogram of aspect ratio *a*/*c* for SS1. **(G)** Variogram of surface area for AS1. **(H)** Variogram of surface area for AS2. **(I)** Variogram of surface area for AS3. **(J)** Variogram of surface area for AS4. **(K)** Variogram of surface area for AS5. **(L)** Variogram of surface area for SS1. The dashed-curves show point-wise 2.5 and 97.5% quantiles for the distribution of *V*_*m*_(*t*), while the red curves indicate the observed variograms for the five asymmetric (AS1–AS5) and one symmetric (SS1) synapses.

Figure 9 shows the variograms for size and shape of the synaptic vesicles. In this case the variograms fall within the 2.5 and 97.5% quantiles under the null model. This indicates that there is not a significant interaction between the size and shape characteristics for pairs of vesicles.

## 6. Discussion

Our results in the model mouse demonstrate that in asymmetric and symmetric synapses the synaptic vesicles are not spherical but instead appear to be oblate and cigar-shaped ellipsoids, respectively. One speculation for the reason of non-sphericity could be the electrostatic polarity of the bilayer membrane of the synaptic vesicles. It could also be because of the electrostatic gradient through the synapse, which pulls the synaptic vesicles toward the active zone. Uchizono (1965) claimed that the ellipsoidal synaptic vesicles can be found in inhibitory synapses and the excitatory synapses have vesicles of spherical shape. Walberg (1968), maintained that the elongated or flat vesicles were merely the result of aldehyde fixation, while Uchizono (1968) provided more evidence that they were related to inhibitory synapses. Ushizono's idea was strengthened by Fukami (1969), who demonstrated that after osmium tetroxide fixation without initial aldehyde fixation, flat synaptic vesicles exist in a proportion of the synapses in the spinal cord of frog and snake. Our results indicate that the synaptic vesicles in excitatory synapses appear to be of oblate shape ellipsoids and in inhibitory synapses cigar shape ellipsoids. Since the vesicles are not spherical it makes sense to consider their orientations as represented by the orientation vectors of the ellipsoids fitted to the vesicles. We found that the majority of the synaptic vesicles in asymmetric synapses are oriented with their shortest orientation vector perpendicular to the shortest straight path connecting their center to the active zone. The majority of the synaptic vesicles in symmetric synapses are oriented with their shortest and longest orientation vectors perpendicular to the shortest straight path connecting their center to the active zone. This could be in order to minimize resistance to movement toward the active zone. It could also be the case that the vesicles rotate around the short axis toward the active zone in asymmetric synapses, while they move along the middle axis in symmetric synapses.

We analyzed the spatial interaction of the synaptic vesicles by employing spatial point process methods. The results showed that there exist repulsive interactions between the neighboring synaptic vesicles in asymmetric synapses. This may be caused by electrostatic repulsions between the negatively charged synaptic vesicles. On the contrary, in symmetric synapse, there is no repulsive interactions between the neighboring synaptic vesicles beyond non-overlap. In larger distances the results showed aggregation of synaptic vesicles in both types of synapses, which may be due to their reserve pool. given area of layer 5 in a mouse somatosensory barrel cortex in 50–100 μm thick slices. Edge corrections were used to account for the effects of neurons located outside the box. In our context the observation region is delimited by the membrane of the pre-synaptic neuron and edge corrections are not relevant as we do not expect vesicles inside one synapse to interact with vesicles in other synapses.

Anton-Sanchez et al. (2014) used a random sequential adsorption (RSA) point process as a null model for testing hypotheses of no interaction within chemical synapses. This type of point process is simulated by sequentially adding points representing synapses until a pre-specified number of points is obtained while rejecting points whenever a proposed new synapse intersects previously added synapses. This point process model is thus defined by its construction. One issue with this approach is that the probabilistic properties of the resulting point process are not well understood. We instead define a null model of no interaction as follows: the number of vesicles inside a synapse coincides with the observed number of vesicles. Next, vesicles appear independently of each other except that they respect the physical constraints of no overlap with other vesicles, mitochondria, lysosomes, or the synapse membrane. While the definition of the model is straightforward the simulation of it is not. Simulations can, however, be obtained using a Markov Chain Monte Carlo algorithm.

Comparing the *K*-function of the observed data and simulations from the null model indicate that there is a clear repulsion between the synaptic vesicles in addition to repulsion due to the non-overlaping constraint. The variogram of the orientations of neighboring vesicles further showed that there are correlations between orientations of vesicles for a wide range of inter-vesicle distances. Thus, the ensemble of vesicles is aligned with respect to their orientations.

We considered five asymmetric synapses and one symmetric synapse and it is striking that the findings for the five synapses were consistent and different from one symmetric synapse. However, in this study we are looking at data from only one mouse and therefore further data need to be collected in order to check whether our results can be produced for other mouse. Also, manual annotation of the FIB-SEM images is extremely time consuming. It would therefore be of great interest to develop an automatic procedure for annotation of synapses and their interiors in FIB-SEM images.

## Author contributions

MK carried out all analyses and wrote the first draft of the manuscript. JS formulated the problem of analysing shape and interaction for synaptic vesicles. RW provided input regarding the statistical analyses and the point process methodology. MK, RW, and JS jointly wrote the final version of the manuscript.

## Funding

VILLUM FOUNDATION and Danish Council for Independent Research|Natural Sciences Grant 12124675.

### Conflict of interest statement

The authors declare that the research was conducted in the absence of any commercial or financial relationships that could be construed as a potential conflict of interest.
